# Impact of Remote Service Model on Social Functioning of Chronic Mental Illness Patients in Community Psychiatric Rehabilitation Centers during the COVID-19 Pandemic: A Retrospective Cohort Study

**DOI:** 10.1192/j.eurpsy.2026.10942

**Published:** 2026-07-31

**Authors:** Y.-C. Wu, N.-Y. Teng

**Affiliations:** Taipei City Hospital, Taipei City, Taiwan

## Abstract

**Introduction:**

The COVID-19 pandemic disrupted rehabilitation services for individuals with chronic mental illness, increasing the risk of social functional decline. Community psychiatric rehabilitation centers help maintain functional abilities, but interruptions can accelerate deterioration. During closures, some centers adopted remote rehabilitation to sustain activities and engagement, yet its long-term effectiveness in this setting is unclear. This study provides one of the first four-year longitudinal evaluations of remote service provision in such centers, spanning the pre-pandemic, peak-pandemic, and recovery phases.

**Objectives:**

To compare changes in social functioning, measured by the Personal and Social Performance Scale (PSP), between patients receiving remote services and those without remote services during the COVID-19 pandemic.

**Methods:**

A four-year retrospective cohort study was conducted on 139 patients with chronic mental illness from two community psychiatric rehabilitation centers. PSP was assessed every six months. During service interruptions, one center implemented remote rehabilitation while the other did not. Patients were categorized into three groups: remote-service participants (A1), non-participants in the same center (A2), and patients from a center without remote services (B). Longitudinal PSP score differences among groups were analyzed using Generalized Estimating Equations (GEE).

**Results:**

The study included 139 patients (mean age = 63.95 ± 11.87 years; 48.2% male; 79.1% schizophrenia). PSP scores showed divergent trends during the pandemic: the remote-service group (A1) increased even at the pandemic peak, while B declined sharply. By T7 (mid-2023), A1 exceeded baseline levels, whereas A2 and B showed only partial recovery (Table 1).

**Table 1.** GEE estimates (β, SE, p) of PSP social functioning across pandemic periods by group Table 1. Long description.

Pandemic period (ref: T0)	A1, N=25, 216 obs.			A2: N=54, 448 obs.			B: N=59, 472 obs.		
	β	SE	P VALUE	β	SE	P VALUE	β	SE	P VALUE
T1	0.60	0.46	0.195	0.63	0.40	0.113	–0.61	0.20	0.002
T2	1.95	0.87	0.024	1.07	0.47	0.022	–1.77	0.56	0.002
T3	2.70	1.42	0.057	0.52	0.91	0.569	–1.86	1.04	0.075
T4	8.02	2.06	<0.001	1.37	1.25	0.271	–5.55	1.50	<0.001
T5	10.81	2.43	<0.001	4.91	2.11	0.020	–1.08	1.25	0.390
T6	7.83	1.92	<0.001	0.34	0.95	0.723	–4.54	1.34	<0.001
T7	8.61	2.06	<0.001	0.70	1.06	0.506	–1.32	1.31	0.312
T8	8.65	2.19	<0.001	–0.06	1.34	0.964	–0.97	1.22	0.424

**Conclusions:**

Remote service provision during the COVID-19 pandemic was linked to better preservation and recovery of social functioning in chronic mental illness patients in community psychiatric rehabilitation. Findings support integrating remote rehabilitation into routine care to ensure continuity and protect functional outcomes in future public health crises.

**Disclosure of Interest:**

None Declared

## Long descriptions

### Table 1. Long description

The table presents beta, S E, and P V A L U E for three groups across eight pandemic periods (T 1 to T 8), using T 0 as the reference.

* Group A 1 (N equals 25, 216 observations): Shows a significant positive trend in beta values starting from T 2 (beta 1.95, p 0.024) and peaking at T 5 (beta 10.81, p less than 0.001). Values remain high and significant through T 8 (beta 8.65, p less than 0.001).

* Group A 2 (N equals 54, 448 observations): Shows significant positive beta values at T 2 (beta 1.07, p 0.022) and T 5 (beta 4.91, p 0.020). Other periods like T 6 (beta 0.34) and T 8 (beta minus 0.06) are not statistically significant.

* Group B (N equals 59, 472 observations): Shows predominantly negative beta values. Significant decreases are noted at T 1 (beta minus 0.61, p 0.002), T 2 (beta minus 1.77, p 0.002), T 4 (beta minus 5.55, p less than 0.001), and T 6 (beta minus 4.54, p less than 0.001).

Navigate back to Table 1..

